# Differential Regulation of LPS-Mediated VE-Cadherin Disruption in Human Endothelial Cells and the Underlying Signaling Pathways: A Mini Review

**DOI:** 10.3389/fcell.2019.00280

**Published:** 2020-01-06

**Authors:** Yee Han Chan, Hanis Hazeera Harith, Daud Ahmad Israf, Chau Ling Tham

**Affiliations:** Department of Biomedical Science, Faculty of Medicine and Health Sciences, Universiti Putra Malaysia, Serdang, Malaysia

**Keywords:** VE-cadherin, adherens junction, lipopolysaccharide, endothelial hyperpermeability, tyrosine phosphorylation, internalization

## Abstract

Endothelial cells lining the inner vascular wall form a monolayer that contributes to the selective permeability of endothelial barrier. This selective permeability is mainly regulated by an endothelium-specific adherens junctional protein, known as vascular endothelial-cadherin (VE-cadherin). In endothelial cells, the adherens junction comprises of VE-cadherin and its associated adhesion molecules such as p120, α-catenin, and β-catenin, in which α-catenin links cytoplasmic tails of VE-cadherin to actin cytoskeleton through β-catenin. Proinflammatory stimuli such as lipopolysaccharide (LPS) are capable of attenuating vascular integrity through the disruption of VE-cadherin adhesion in endothelial cells. To date, numerous studies demonstrated the disruption of adherens junction as a result of phosphorylation-mediated VE-cadherin disruption. However, the outcomes from these studies were inconsistent and non-conclusive as different cell fractions were used to examine the effect of LPS on the disruption of VE-cadherin. By using Western Blot, some studies utilized total protein lysate and reported decreased protein expression while some studies reported unchanged expression. Other studies which used membrane and cytosolic fractions of protein extract demonstrated decreased and increased VE-cadherin expression, respectively. Despite the irregularities, the results of immunofluorescence staining are consistent with the formation of intercellular gap. Besides that, the overall underlying disruptive mechanisms of VE-cadherin remain largely unknown. Therefore, this mini review will focus on different experiment approaches in terms of cell fractions used in different human endothelial cell studies, and relate these differences to the results obtained in Western blot and immunofluorescence staining in order to give some insights into the overall differential regulatory mechanisms of LPS-mediated VE-cadherin disruption and address the discrepancy in VE-cadherin expression.

## Introduction

Endothelial cells form a continuous inner lining which controls the movement of plasma proteins and other circulating substances between blood plasma and the interstitial space. This selective vascular permeability is attributed to transcellular and paracellular pathways; in which the latter plays a predominant role. Transcellular pathway is mediated by the utilization of vesicular system which brings the internalized proteins from the apical side to the basal side of plasma membrane and subendothelial sites (Dejana et al., 1999). Paracellular pathway is mediated by the localization of the adhesive cell-cell junctional proteins which is further categorized into tight junction, adherens junction, and gap junction.

Tight junction and adherens junction are transmembrane proteins which form a zig-zag mosaic along the interendothelial cell borders. In contrast, gap junction is made up of channel which allows the passage of small molecules and cell-cell communication (Komarova et al., 2017). In endothelial cells, tight junctions consist of occludin and claudin which interact with intracellular zonula occludens (ZO) 1, 2, or 3. On the other hand, endothelial adherens junctions are made up of cadherin-catenin protein complex which includes VE-cadherin, p120, α-catenin, and β-catenin (Vestweber, 2008). Among these, adherens junction is considered to be the vital junctional protein in the regulation of endothelial permeability.

It is important to highlight that the term “VE-cadherin” used in this mini review refers to VE-cadherin, a member of endothelial adherens junctional protein as opposed to some studies which referred to it as adherens junction. This is because endothelial adherens junction is made of VE-cadherin and its associated catenin protein molecules including p120, α-catenin, and β-catenin, in which α-catenin links cytoplasmic tails of VE-cadherin to actin cytoskeleton through β-catenin (Vestweber, 2008). This endothelial adherens junction undergoes a series of dynamic assembly, disassembly, and remodeling through the interaction with the actin cytoskeleton in response to mechanical and chemical stimuli in both physiological and pathological conditions (Dejana et al., 1999).

Endothelial hyperpermeability is a hallmark of sepsis as a consequence of bacterial infection. As endothelial permeability is mainly attributed to adherens junction, numerous studies had been performed to investigate the expression of VE-cadherin in response to external stimuli such as LPS which is found in the outer membrane of Gram-negative bacteria. However, the experimental approaches and outcomes of these studies were not consistent. In particular, some studies reported decreased VE-cadherin expression while others did not. Despite these irregularities, the majority of studies consistently reported the formation of intercellular gap as indicated by immunofluorescence staining of VE-cadherin. Therefore, this review aims to summarize the differences in experimental approaches used in previous *in vitro* and *ex vivo* studies, and relate these differences to the results obtained in Western blot and immunofluorescence staining in order to clarify the ambiguous mechanisms of LPS-mediated VE-cadherin disruption and explain the discrepancy in VE-cadherin expression.

## Mechanisms of LPS-Induced VE-Cadherin Disruption

As a cadherin member of endothelial adherens junction, the disruption of VE-cadherin can be due to tyrosine phosphorylation, internalization, endocytosis, and lysosomal degradation. Ambiguously, these reported modes of disruption lead to two major outcomes which are reduced or unchanged VE-cadherin expression. The experimental approaches, results obtained and signaling pathways involved in LPS-mediated VE-cadherin disruption in various human endothelial cell models are summarized in Table 1.

**Table 1:** Experimental approaches, results obtained, and signaling pathways involved in LPS-mediated VE-cadherin disruption in various human endothelial cell models.

**Human endothelial cell model**	**LPS strain; concentration; induction duration**	**Cell fraction used**	**Protein expression**		**Mode of disruption**	**Signaling pathways involved**	**References**
			**Western blot**	**Immunofluorescence staining**			
Human Umbilical Vein Endothelial Cell (HUVEC)	N/A; 1 μg/mL; 1 h	Total protein	↓	Intercellular gap formation	Reduced protein expression	Activation of MLCK/MLC pathway	Huang et al., 2015a
	*E.coli* O55:B5; 1 μg/mL; 4 h	Total protein	↓	N/A	Reduced protein expression	Tyrosine phosphorylation of VE-cadherin at Y658 and Y731	Chen J. et al., 2015
	*E.coli* O55:B5; 1 μg/mL; 6 h	Total protein	↓	N/A	Reduced protein expression	Decreased phosphorylation of AMPK, ACC, and LKB1	Yang et al., 2017
	*E.coli* O55:B5; 1 μg/mL; 12 h	Total protein	↓	N/A	Reduced protein expression	Increased level of TNF-α and IL-1β	Deng et al., 2019
	N/A; 1 μg/mL; 12 h	Total protein	↓	N/A	Reduced protein expression	Decreased expression of HSPA12B and increased phosphorylation of MLC	Kang et al., 2016
	N/A; 1 μg/mL; 24 h	Total protein	↓	Intercellular gap formation	Reduced protein expression	Increased expression of ROCK	Xie et al., 2015
	N/A; 1 μg/mL; N/A	Total protein	↓	N/A	Reduced protein expression	Activation of p38 MAPK, ERK, JNK, and NF-κB pathways	Chen et al., 2019
	N/A; 10 μg/mL; 4 h	Total protein	↓	N/A	Reduced protein expression	Synergistic effect of LPS and urocortin, followed by dissociation of VE-cadherin and endocytosis	Wan et al., 2012
	N/A; 20 μg/mL; 72 h	Total protein	↓	Intercellular gap formation	Reduced protein expression	ALK5 activity-dependent endothelial-mesenchymal transition-like process	Echeverría et al., 2013
	*E.coli* O55:B5; N/A; 24 h	Total protein	↓	Intercellular gap formation	Reduced protein expression	Activation of p38 MAPK, ERK1/2, and Akt pathway	Tang et al., 2016
	N/A; N/A; 24 h	Total protein	↓	N/A	Reduced protein expression	Activation of p38 MAPK pathway	Chu et al., 2016
	N/A; N/A; N/A	Total protein	↓	Intercellular gap formation	Reduced protein expression	TRPC1-induced Ca^2+^ influx	Pang et al., 2011
	N/A; 0.1 μg/mL; 30 and 90 min	Total protein	↔	Intercellular gap formation	Phosphorylation of VE-cadherin at Y658	Increased expression and phosphorylation of caveolin-1 at Y14; SRC phosphorylation at Y416	Pan et al., 2015
	N/A; 0.5 μg/mL; 2 h	Total protein	↔	Intercellular gap formation	Dissociation of α-catenin from VE-cadherin	Activation of xanthine oxidase, ROS production, SHP2 inactivation, Frk activation, and tyrosine phosphorylation of VE-cadherin	Chattopadhyay et al., 2017
	N/A; 10 μg/mL; 12 h	Total protein	↔	N/A	Phosphorylation of VE-cadherin	Activation of Ras/Raf/MEK/ERK	Haidari et al., 2012
Human Pulmonary Microvascular Endothelial Cell (HPMEC)	N/A; 0.1 μg/mL; 1, 2, 4, 8, 12 h	Total protein	↓	N/A	Reduced protein expression	Paxillin tyrosine phosphorylation at Y31 and Y118 leading to VE-cadherin phosphorylation at Y658	Fu et al., 2015
	*E.coli* O111:B4; 0.1 μg/mL; 6 h	Total protein	↓	N/A	Reduced protein expression	Activation of Ang2 and Ras	He et al., 2015
	N/A; 0.1 μg/mL; 6 h	Total protein	↓	Intercellular gap formation	Reduced protein expression	Activation of RhoA/Rac1 pathway and upregulation of caveolin-1	Yang Y. et al., 2015
	N/A; 0.1 μg/mL; 6 h	Total protein	↓	Intercellular gap formation	Reduced protein expression	Upregulation of caveolin-1	Chen Q. H. et al., 2015
	*E.coli* O55:B5; 0.1 μg/mL; 24 h	Total protein	↓	N/A	Reduced protein expression	Inhibition of Sirt3/AMPK pathway and upregulation of Ang2 expression	Chen et al., 2018
	N/A; 1 μg/mL; 24 h	Total protein	↓	Intercellular gap formation	Reduced protein expression	Activation of Rho/MLC and NF-κB pathways	Xu et al., 2019
	N/A; 1 μg/mL; 24 h	Total protein	↓	Intercellular gap formation	Reduced protein expression	Activation of ROCK/MLC, NF-κB, and p38 pathways	Wang et al., 2018
	*E.coli* O55:B5; 2 μg/mL; 24 h	Total protein	↓	N/A	Reduced protein expression	Increased expression of HMGB1 and TLR4, and activation of NF-κB pathway	Zhang et al., 2018
	*E.coli* O111:B4; 0.1 μg/mL; 12 h	Total, membrane and cytosolic protein	Total protein ↔; membrane protein ↓; cytosolic protein ↑	Intercellular gap formation	Internalization	Activation of GEF-H1/RhoA/ROCK signaling pathway	Huang et al., 2015b
	N/A; 0.1 μg/mL; 24 h	Total protein	↔	N/A	Dissociation of β-catenin and γ-catenin from VE-cadherin	Activation of Erk/p38/Src pathway	Liu et al., 2017
	*E.coli* O111:B4 and O55:B5; 1 μg/mL; 6 h	Total, membrane and cytosolic protein	Total protein ↔; membrane protein ↓; cytosolic protein ↑	Intercellular gap formation	Internalization	VE-cadherin translocation mediated by increased Rab5a activity and expression	Yang J. et al., 2015
Human Pulmonary Artery Endothelial Cell (HPAEC)	N/A; 1 μg/mL; 24 h	Total protein	↓	Intercellular gap formation	Reduced protein expression	Activation of NF-κB, p38 MAPK, JNK, and ERK pathways	He et al., 2019
Human Dermal Microvascular Endothelial Cell (HDMEC)	*E.coli* O111:B4 0.1 μg/mL; 2 h	Total protein	↔	Intercellular gap formation	Reduced VE-cadherin adhesion	Decreased intracellular cAMP leading to inactivation of small GTPase Rac1	Schlegel et al., 2009

*N/A, not applicable*.

### Reduced Protein Expression

To date, there are sufficient evidences that favor the contribution of transcellular and paracellular pathways in the regulation of vascular permeability; predominated by the latter. Both pathways have recently been found to be interrelated in the regulation of LPS-induced endothelial hyperpermeability which involves the degradation of VE-cadherin. There are several modes of action and pathways mediating the degradation of VE-cadherin in LPS-stimulated human endothelial cells.

#### Synergistic Effect

In the manifestation of vascular hyperpermeability, LPS triggers the release of proinflammatory cytokines which subsequently act synergistically with LPS in the disruption of junctional integrity. Previous studies revealed that a member of corticotrophin-releasing hormone (CRH), namely urocortin (Ucn1), is released significantly upon LPS stimulation. It acts as a peripheral proinflammatory cytokine which exacerbates LPS-induced endothelial hyperpermeability (Wan et al., 2012). Upon LPS stimulation, Ucn1 triggers the phosphorylation of protein kinase D via CRF2 receptor and activates the downstream target heat shock protein 27 (HSP27) through corticotrophin-releasing hormone receptor 2 (CRHR2). This results in β-catenin phosphorylation at serine residue S552, accompanied by the dissociation of VE-cadherin (Wan et al., 2012).

#### Tyrosine Phosphorylation of VE-Cadherin

The tyrosine phosphorylation of VE-cadherin was thought to be the major mode of VE-cadherin degradation. Fu et al. (2015) demonstrated that the phosphorylation of tyrosine residues Y31 and Y118 of paxillin, a focal adhesion adaptor protein, eventually leads to VE-cadherin tyrosine phosphorylation at Y658. This is in line with a study by Chen J. et al. (2015) which also reported tyrosine phosphorylation of VE-cadherin at residues Y658 and Y731. As a consequence of phosphorylation, VE-cadherin dissembles from the endothelial adherens junction.

#### Rho/ROCK/MLC Pathway

Several studies suggest the involvement of the MyD88-independent Rho/ROCK/MLC pathway in LPS-induced endothelial hyperpermeability. Rho/ROCK/MLC pathway comprises of a series of signaling molecules such as Rac1 (Yang Y. et al., 2015), RhoA (Yang Y. et al., 2015), Rho (Xu et al., 2019), Rho-associated coiled protein kinase 1/2 (ROCK1/2) (Xie et al., 2015; Wang et al., 2018), myosin light chain phosphatase (MLCP) (Wang et al., 2018; Xu et al., 2019), myosin light chain kinase (MLCK) (Huang et al., 2015a; Yang Y. et al., 2015; Wang et al., 2018; Xu et al., 2019), and myosin light chain (MLC) (Huang et al., 2015a; Yang Y. et al., 2015; Kang et al., 2016; Wang et al., 2018; Xu et al., 2019). Activation of these molecules has been demonstrated to result in MLC phosphorylation and subsequent VE-cadherin degradation (Huang et al., 2015a; Yang Y. et al., 2015; Kang et al., 2016; Wang et al., 2018; Xu et al., 2019). Yang Y. et al. (2015) reported the inhibition of Rac1 and activation of RhoA which activate Rho during the initiation of LPS signaling. Subsequently, the study of Wang et al. (2018) demonstrated the activation of ROCK1/2 which is located downstream of Rho, followed by the inactivation of myosin light chain phosphatase (MLCP). This is supported by Xu et al. (2019) who also reported the activation of Rho and inactivation of MLCP by phosphorylating myosin phosphatase target subunit 1 (MYPT1), a regulatory subunit of MLCP. Following MLCP inactivation, MLCK is switched on and becomes activated, resulting in the phosphorylation of MLC (Huang et al., 2015a; Yang Y. et al., 2015; Kang et al., 2016; Wang et al., 2018; Xu et al., 2019). This is in agreement with the study of Kang et al. (2016) which reported MLC phosphorylation as a result of downregulation of heat shock protein A12B (HSPA12B). Although these studies did not investigate the tyrosine phosphorylation of VE-cadherin, MLC phosphorylation has been demonstrated to result in the degradation of VE-cadherin (Huang et al., 2015a; Xie et al., 2015; Yang Y. et al., 2015; Kang et al., 2016; Wang et al., 2018; Xu et al., 2019).

#### Transcellular Pathway via Caveolae-Mediated Endocytosis

Interestingly, the study of Yang Y. et al. (2015) demonstrated the involvement of transcellular pathway in VE-cadherin degradation as indicated by LPS-induced upregulation of caveolin-1 and the subsequent formation of caveolae, which paralleled Chen Q. H. et al.'s (2015) study whereby they reported the increased expression and phosphorylation of caveolin-1. These findings suggest that transcellular pathway via vesicular action, mediates the degradation of VE-cadherin through endocytosis. The unbound VE-cadherin is internalized into intracellular compartment through the invaginated caveola forming early endosome upon the endocytosis of VE-cadherin (Chen Q. H. et al., 2015; Yang Y. et al., 2015). Meanwhile, tyrosine kinase Src binds to the VE-cadherin-positive early endosome by interacting with endosome regulating protein, namely β-arrestin (Chichger et al., 2016). The tethering of Src kinase to endosome via β-arrestin, activates Src by phosphorylating it at residue Y416 (Chichger et al., 2016). As a result, endosome binding protein, p18, acts as a substrate to drive its own phosphorylation at residue 40 (Chichger et al., 2014). Rab GTPase is a family of small GTPase that regulates and coordinates vesicle trafficking. Rab GTPases are categorized into pro-degradation Rab (Rab7 and Rab9) and pro-recycling Rab (Rab4 and Rab5a) (Chichger et al., 2016). The recruitment of p18 promotes the interaction between the early endosome containing dissociated VE-cadherin with pro-degradation Rab7 instead of pro-recycling Rab11. This ultimately leads to late endosomal or lysosomal degradation of VE-cadherin (Chichger et al., 2014).

#### SOC-Mediated Pathway

Similarly, the study of Pang et al. (2011) demonstrated the degradation of VE-cadherin by transient receptor potential protein C1 (TRPC-1)-induced calcium influx. Previous studies also identified TRCP-1 as an essential component of store-operated calcium channel (SOC) which enables the influx of calcium ions in endothelial cells, contributing to VE-cadherin dissociation and cytoskeleton remodeling (Sandoval et al., 2001; Tiruppathi et al., 2006; Vandenbroucke et al., 2008).

#### Sirt3/AMPK Pathway

Also, another study demonstrated that the inhibition of Sirt3/adenosine monophosphate-activated protein kinase (Sirt3/AMPK) pathway can result in the degradation of VE-cadherin (Chen et al., 2018). In previous studies, AMPK has been identified as an enzyme that regulates Sirt3 by promoting the expression of peroxisome-activator receptor coactivator-1α (PGC-1α) as well as oxidative stress in autophagy (Palacios et al., 2009; Giralt et al., 2011; Kahn et al., 2015; Pillai et al., 2015). This is in line with the study of Yang et al. (2017) which also reported a notable decrease in liver kinase B (LKB1), followed by decreased phosphorylation of AMPK and its downstream kinase, namely acetyl coenzyme A carboxylase (Acc) at serine residue 79.

#### Ang2/Ras Pathway

Apart from the involvement of Sirt3/AMPK pathway, Chen et al. (2018) also demonstrated that upregulation of angiotensin 2 (Ang2) is associated with VE-cadherin degradation in response to LPS induction. Increased Ang2 levels results in the activation of rennin-angiotensin system (Ras) which is known to be involved in key events of inflammatory process (Yusuke et al., 2003). Similarly, He et al. (2015) also explored the activation of Ras mediated by increased level of Ang2, which eventually lead to the degradation of VE-cadherin.

#### Secretion of HMGB1

During LPS challenge, an inflammatory cytokine—high motility group box protein 1 (HMGB1) is secreted by immune cells to drive the pathologic inflammation by activating the inflammatory signaling pathway through Toll-like receptor 4 (TLR4). This is supported by the study of Zhang et al. (2018) which demonstrated the increased secretion of HMGB1 and expression of TLR4 receptor in LPS-stimulated Human Pulmonary Microvascular Endothelial Cell (HPMEC).

#### Endothelial-Mesenchymal Transition-Like Process (EndMT)

Interestingly, a study demonstrated VE-cadherin degradation in LPS-stimulated endothelial cells due to activin receptor-like kinase 5 (ALK5) activity-dependent endothelial-mesenchymal transition-like process (Echeverría et al., 2013). This study clearly showed that LPS is able to activate transforming growth factor-beta (TGF-β) which subsequently activate its own receptor ALK5. As a consequence, endothelial fibrosis occurs, followed by subsequent endothelial-mesenchymal transition-like process. This process is accompanied by the downregulation of endothelial biomarkers including VE-cadherin and CD31, and the upregulation of α-smooth muscle actin, which ultimately leads to endothelial permeability (Echeverría et al., 2013).

#### Other Pathways

Furthermore, there are numerous studies which suggest the participation of nuclear factor kappa-light chain enhancer of activated B cells (NF-κB) (Wang et al., 2018; Zhang et al., 2018; Chen et al., 2019; He et al., 2019; Xu et al., 2019), p38 mitogen-activated protein kinase (p38 MAPK) (Chu et al., 2016; Tang et al., 2016; Wang et al., 2018; Chen et al., 2019; He et al., 2019), extracellular receptor kinase (ERK) (Tang et al., 2016; Chen et al., 2019; He et al., 2019), c-Jun N-terminal kinase (JNK) (Chen et al., 2019; He et al., 2019), and protein kinase B (PKB or AKT) (Tang et al., 2016) pathways in LPS-mediated VE-cadherin degradation in human endothelial cells. In addition, LPS had also been reported to increase the level of proinflammatory cytokines such tumor necrosis factor-alpha (TNF-α) and interleukin-1 beta (IL-1β), resulting in the downregulation of VE-cadherin (Deng et al., 2019). However, a detailed investigation on the involvement of these signaling pathways is still lacking.

#### Fate of β-Catenin

Surprisingly, although tyrosine phosphorylation leads to the downregulation of VE-cadherin, the unbound β-catenin was found to be accumulated in both cytosol and nucleus. This may be due to the inactivation of β-catenin degrading enzyme GSK-3β. Subsequently, β-catenin translocates into the nucleus and interacts with transcription factor of TCF/LEF family, contributing to the transcription of downstream genes such as vascular endothelial growth factor (VEGF) which further augments the vascular hyperpermeability (Huang et al., 2015a).

#### Overall Mechanisms

**Figure 1:**
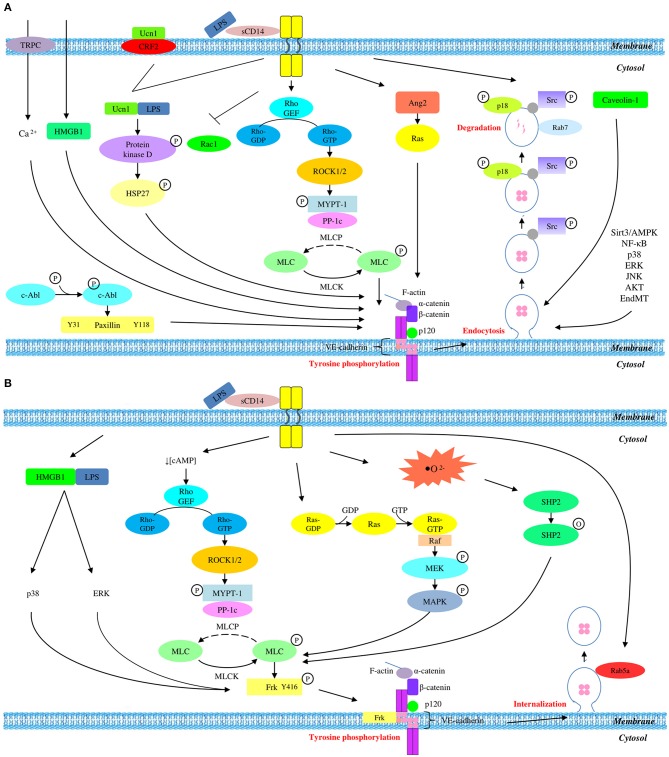
Overall mechanisms of LPS-mediated VE-cadherin disruption which result in **(A)** reduced protein expression and **(B)** unchanged protein expression.

Although VE-cadherin is a part of endothelial adherens junction which is known to be a coordinator of paracellular pathway, its protein expression is partly regulated via transcellular route. In short, it is concluded that LPS mediates the disruption of VE-cadherin through a series of transcellular events, starting with tyrosine phosphorylation of VE-cadherin, followed by its disassembly from endothelial adherens junction, internalization from membrane into cytosol via endocytosis, and degradation by lysosome. This contributes to the degradation of endogenous VE-cadherin which eventually leads to the disruption of adherens junction and endothelial hyperpermeability. A summary of LPS-mediated VE-cadherin degradation is presented graphically in Figure 1A.

### Unchanged Protein Expression

The compromised endothelial integrity as a result of VE-cadherin disruption can be attributed to tyrosine phosphorylation which subsequently leads to internalization. As opposed to most studies which reported reduced VE-cadherin expression, there are several studies which reported unchanged total VE-cadherin expression. Studies which reported unchanged VE-cadherin expression utilized total protein lysate as well as membrane and cytosolic fractions of protein extract for the examination of VE-cadherin disruption in response to LPS stimulation. It is speculated that the reason behind the use of this experimental approach is the phosphorylation-dependent VE-cadherin internalization which translocates VE-cadherin from membrane into cytosol while maintaining the total endogenous protein expression. This process occurs in a series of signaling events, starting with MLC phosphorylation, followed by Src-mediated tyrosine phosphorylation of VE-cadherin and its internalization into cytosol.

#### MLC Phosphorylation

In this review, GEF/Rho/ROCK and Ras/Raf/MEK/MERK pathways are identified as the pathways leading to MLC phosphorylation. Schlegel et al. (2009) demonstrated an increased level of cyclic adenosine monophosphate (cAMP), followed by the inactivation of a Rho GTPase, namely Rac1. As Rac1 is a downstream modulator of Rho, this finding is in line with a report by Huang et al. (2015b) which implicated a role for GEF/Rho/ROCK pathway in MLC phosphorylation. Therefore, the findings of these two studies will be linked and discussed as a whole GEF/Rho/ROCK pathway in this section. During LPS stimulation, cAMP level is decreased as an initial sign of LPS insult (Schlegel et al., 2009). This activation process immediately leads to the activation of Rho guanine exchange factor (Rho GEF), followed by its downstream protein RhoA. The active Rho-GTP is now capable of activating ROCK while inhibiting Rac1 as indicated in the study of Schlegel et al. (2009). Following ROCK activation, phosphorylation of myosin phosphatase target subunit 1 (MYPT1) occurs and drives MLCP inactive. The inactivation of MLCP immediately activates and switches MLCK on, resulting in MLC phosphorylation. Being consistent with aforementioned findings, the study of Haidari et al. (2012) also demonstrated that MLC phosphorylation occurs via Ras/Raf/MEK/ERK pathway. This pathway is also known as MAPK/ERK pathway.

#### Src-Mediated Tyrosine Phosphorylation of VE-Cadherin

It is well-established that MLC phosphorylation leads to Src phosphorylation and activation. This is supported by Pan et al. (2015) who reported Src phosphorylation occurs at tyrosine residue Y416. Similarly, Liu et al. (2017) demonstrated that the secretion of HMGB1 leads to Src phosphorylation via p38 and ERK pathways. This is also in agreement with another study which demonstrated Src-mediated tyrosine phosphorylation of VE-cadherin (Chattopadhyay et al., 2017). Specifically, LPS challenge provokes the activation of xanthine oxidase (XO) which catalyzes the production of reactive oxygen species (ROS) (Yang J. et al., 2015). Subsequently, a protein tyrosine kinase, namely Src homology region 2-containing phosphatase (SHP2) is inactivated by its oxidation at cystein residue 456, resulting in its dissociation from VE-cadherin. Consequently, a Src family of protein tyrosine kinase, namely Frk is phosphorylated and activated. This active Fyn-related kinase (Frk) is then recruited to VE-cadherin and β-catenin. Eventually, tyrosine phosphorylation of VE-cadherin, α-catenin, β-catenin, and p120 catenin occur, particularly VE-cadherin and α-catenin more robustly (Chattopadhyay et al., 2017).

#### VE-Cadherin Internalization

The tyrosine phosphorylation of various members of adherens junction eventually leads to the disassembly of VE-cadherin from endothelial adherens junction. The unbound VE-cadherin is then internalized into the intracellular compartment through early endosome which is invaginated from its own plasma membrane (Chattopadhyay et al., 2017). This is in agreement with a study by Yang J. et al. (2015) which revealed the localization of Rab5a to the early endosomes during VE-cadherin internalization.

#### Overall Mechanisms

LPS stimulation triggers tyrosine phosphorylation of VE-cadherin which drives its internalization into the intracellular compartment, followed by the recruitment of pro-recycling Rab5a GTPase which promotes the recycling of internalized VE-cadherin back to membrane. This series of event thereby endorses the unchanged expression of total VE-cadherin in LPS-mediated endothelial dysfunction. A summary of LPS-mediated VE-cadherin internalization is presented graphically in Figure 1B.

## Discussion

Due to variations in the experimental designs used in previous studies, it is challenging to draw a conclusion for the inconsistent data on VE-cadherin expression. However, our findings suggest that the inconsistent reports on the expression of VE-cadherin may be due to the variation in the concentration of LPS used to induce the cells. From Table 1, most studies that used <1 μg/mL LPS failed to demonstrate any reduction in VE-cadherin expression. In contrast, most studies which demonstrated VE-cadherin downregulation induced the cells with 1 μg/mL LPS or higher concentrations. These findings may be due to the effects of different LPS concentrations on the expression of VE-cadherin through PI3K/Akt pathway as reported by Zheng et al. (2018). Specifically, Zheng et al. (2018) reported that lower concentration of LPS (0.01 μg/mL) promotes VE-cadherin expression while higher concentrations (0.1, 1, 10, and 100 μg/mL) reduce VE-cadherin expression. Based on these studies, we propose that VE-cadherin would most likely be downregulated if the cells are exposed to higher concentrations of LPS.

Besides that, the inconsistent reports on the expression of VE-cadherin could also be attributed to the duration of LPS induction. Table 1 shows that most studies that demonstrated reduced VE-cadherin expression, induced the cells with LPS for 6 h or longer. In contrast, most studies that reported unchanged VE-cadherin expression, induced the cells with LPS for <6 h. Therefore, these findings suggest that longer duration of LPS induction may be required to trigger the mechanism for downregulation of VE-cadherin. In particular, VE-cadherin downregulation may result from its internalization through endocytic trafficking. This process may be regulated by Rab family of small GTPase which can be categorized into pro-recycling Rab (Rab4 and Rab5a) and pro-degradation Rab (Rab7, Rab9, and Rab11) (Chichger et al., 2016). The latter has been shown to be important for the degradation of internalized VE-cadherin in the early endosomes. The recruitment and binding of the pro-degradation Rab will signal the internalized VE-cadherin to be transported to the lysosomes for degradation. Ultimately, the total and membrane VE-cadherin are expected to decrease while cytosolic VE-cadherin is expected to revert to the basal level. Meanwhile, the binding of pro-recycling Rab will signal the internalized VE-cadherin to be recycled back to the membrane. As such, the expression of VE-cadherin in the membrane and the cytosol would revert to the basal levels, and consequently the total VE-cadherin would appear unchanged. This indicates that the binding of different Rab proteins may also influence the expression of VE-cadherin following LPS induction. Taken together, the assessment of VE-cadherin expression in different fractions of protein lysate (total, membrane, and cytosolic fractions) at one time point only could lead to misinterpretation of the observed changes in VE-cadherin expression following LPS induction. Therefore, it is worthwhile to examine VE-cadherin expression following LPS induction in different fractions of protein lysate and at several time points. Since the role of Rab GTPase in human endothelial cells remains unexplored, studies employing recommended experimental designs would offer more insights into the mechanisms of VE-cadherin regulation.

Another factor that may contribute to the inconsistencies in VE-cadherin expression is the serotype of LPS. As shown in Table 1, most studies that reported reduction in VE-cadherin expression induced the cells with LPS derived from *Escherichia coli* serotype O55:B5, while studies which induced the cells with LPS derived from *E. coli* serotype O111:B4 reported unchanged protein expression. This discrepancy may be due to the potency of different LPS serotypes. Previously, Watanabe and Jaffe (1993) reported that *E.coli* O55:B5 is more potent than *E.coli* O111:B4 in terms of the production of prostaglandin (PGI_2_) and the suppression of angiotensin converting enzyme (ACE) activity. This is also supported by Gaekwad et al. (2010) who demonstrated the differential production of pro-inflammatory cytokines in response to LPS from different *E.coli* serotypes due to the structural differences of Lipid A.

Despite the discrepancies in the Western blot results, we discovered that the immunofluorescence staining results were consistent. The immunofluorescence staining results based on previous studies demonstrated that VE-cadherin is localized at the cell periphery with consistent formation of intercellular gap at the interendothelial cell borders following LPS stimulation. This observation may be explained by the fact that VE-cadherin disruption, either with or without degradation, begins with the phosphorylation-mediated localization of VE-cadherin at the cell periphery which is then internalized and form endosomes to initiate its transport into the cytosol. Apart from the immunofluorescence staining results, previous studies also consistently suggest that VE-cadherin disruption, either with or without degradation, is initiated by tyrosine phosphorylation of VE-cadherin by Src kinase. This is evidenced by the increased expression of phosphorylated VE-cadherin.

## Conclusion

Endothelial adherens junction containing VE-cadherin plays a vital role in maintaining endothelial barrier integrity via its association with catenin molecules. Although VE-cadherin is a part of adherens junction which serves the paracellular pathway, the disruption of VE-cadherin appears to occur in the transcellular pathway, starting with phosphorylation-dependent internalization, which causes reduced or unchanged protein expression. Specifically, we propose that reduced VE-cadherin expression begins with tyrosine phosphorylation by Src kinase, followed by endocytosis and lysosomal degradation as a result of the recruitment of pro-degradation Rab GTPase. In contrast, unchanged VE-cadherin expression is attributed to phosphorylation-mediated internalization from membrane into cytosol, followed by recycling of VE-cadherin back into the membrane as a result of pro-recycling Rab GTPase binding. It is speculated that the inconsistent observations on the total VE-cadherin expression may be due to the effects of different LPS concentrations, the recruitment of different Rab GTPase, and the induction of LPS derived from different *E.coli* serotypes. Notably, this review article proposes several experimental approaches which will enable further characterization of the mechanisms mediating VE-cadherin disruption which may serve as a guide for future researches.

## Author Contributions

YC contributed to the conceptual idea, performed searching, analyzed data, and wrote the manuscript. CT received a grant for this project, conceived the idea, contributed to the conceptual idea, reviewed the drafts, supervised the writing process, and provided important information for the completion of this manuscript. HH and DI contributed to the conceptual idea, gave suggestions and significantly refined the manuscript.

### Conflict of Interest

The authors declare that the research was conducted in the absence of any commercial or financial relationships that could be construed as a potential conflict of interest.
